# Prominent renal complications associated with *MMACHC* pathogenic variant c.80A > G in Chinese children with cobalamin C deficiency

**DOI:** 10.3389/fped.2022.1057594

**Published:** 2023-01-10

**Authors:** Xiaoyu Liu, Huijie Xiao, Yong Yao, Suxia Wang, Hongwen Zhang, Xuhui Zhong, Yanling Yang, Jie Ding, Fang Wang

**Affiliations:** ^1^ Department of Pediatrics, Peking University First Hospital, Beijing, China; ^2^ Laboratory of Electron Microscopy, Pathological Center, Peking University First Hospital, Beijing, China

**Keywords:** cblC, *MMACHC* gene, variant c.80A>G, renal complications, thrombotic microangiopathy (TMA)

## Abstract

**Objective:**

CblC deficiency, the most common cobalamin metabolic abnormality, is caused by pathogenic variants in the *MMACHC* gene. The renal complications of this disease have been described only in a small number of cases. This study aimed to better delineate renal phenotype and genetic characteristics in Chinese children with cblC defect.

**Methods:**

Children with cblC deficiency who manifested as kidney damage were enrolled. Clinical, renal pathological, and genetic data were reviewed in detail.

**Results:**

Seven cases were enrolled. Ages at disease onset ranged from 9 months to 5 years. All patients presented with hematuria and proteinuria, and 2/7 cases presented with nephrotic syndrome. Renal dysfunction was observed in 4/7 cases. Renal biopsy was performed in 5/7 cases, and all of them had renal thrombotic microangiopathy. Macrocytic anemia was detected in all seven patients. Six out of seven cases had hypertension, and 2/7 cases presented with pulmonary hypertension. Two of them had a mild intellectual disability, and one suffered from epilepsy. Increased urine methylmalonic acid and plasma homocysteine were detected in seven cases, while two patients had normal levels of urine methylmalonic acid at the initial evaluation. After diagnosis, all seven cases were treated with hydroxocobalamin IM. Six cases were followed-up for 3–8 years. After treatments, anemia was the first to be recovered, followed by proteinuria. Renal function recovered after 1 year in two cases, whereas patient 2 progressed to stage 2 chronic kidney disease 13 years after onset. While a case presented with end-stage kidney disease because of late diagnosis, one case died 3 months after disease onset due to giving up treatment. Three *MMACHC* pathogenic variants c.80A > G (8/14), c.609G > A (4/14), and c.658_660delAAG (2/14) were detected in all seven children.

**Conclusion:**

*MMACHC* variant c.80A > G may be associated with prominent renal complications in Chinese cblC patients. Macrocytic anemia and hyperhomocysteinemia are useful clues for patients with hematuria and proteinuria caused by cblC defect. The most frequent renal pathological manifestation is thrombotic microangiopathy. Early diagnosis and treatment resulted in improving renal and hematological signs.

## Introduction

Methylmalonic acidemia (MMA) is the most common inborn error of organic acid metabolism, mainly due to methylmalonyl-CoA mutase (MCM) or its coenzyme cobalamin (vitamin B12) metabolic disorders with an autosomal recessively inherited form. Based on the clinical and biochemical features, MMA can be classified as isolated MMA or combined methylmalonic aciduria and homocystinuria, and the latter consists of five subtypes, i.e., cblC, cblD, cblF, cblJ, and cblX deficiencies. Among the five subtypes, the cblC defect (MIM 277400) is the most common type caused by pathogenic variants in the *MMACHC* gene (1). The onset of this disease is usually in infancy or childhood, with heterogeneous clinical manifestations of failure to thrive, macrocytic anemia, macular degeneration, developmental delay, muscular hypotonia, microcephaly, seizures, and other neurological manifestations. Renal manifestations including thrombotic microangiopathy (TMA), atypical hemolytic uremic syndrome (aHUS), and nephrotic syndrome have been described in cblC deficiency (2–4), and *MMACHC* variant c.271dupA was the most frequent damaged allele in patients with cblC defect and renal TMA (5, 6). The most common *MMACHC* variants vary with ethnicities (7, 8), whereas it is not clear whether the genotypes vary among different ethnicities in children with cblC deficiency and prominent renal involvement. This study aimed to better delineate cblC-associated renal disease and the genotypes.

## Patients and methods

Patients with renal disease related to cblC defect were selected from the registry of pediatric hereditary kidney diseases in China (http://chkd.tiamal.com). The following criteria were used to enroll the patients: (i) admitted to the Department of Pediatrics, Peking University First Hospital during 2012–2019 due to renal manifestations; (ii) elevated urine methylmalonic acid (reference range 0.2–3.6 mg/g creatinine) and plasma total homocysteine (reference range 5–15 mmol/L) during the disease course, without a deficiency of plasma vitamin B12; and (iii) (likely) pathogenic variants in the *MMACHC* gene. Patients were excluded if their ages at disease onset were older than 18 years or their clinical data were incomplete.

Clinical, biochemical, and genetic data were collected retrospectively. The Schwartz_bedside_ equation (9) was used to estimate the glomerular filtration rate. Urine methylmalonic acid was measured by gas chromatography–mass spectrometry (GC–MS) analysis. Plasma total homocysteine was measured by a fluorescence polarization immunoassay. *MMACHC* variants were detected by next-generation sequencing or Sanger sequencing, and segregation analyses were performed for parental DNA samples. Each patient was treated with hydroxocobalamin, betaine, and calcium folinate once diagnosed. Treatment effects and prognoses were followed-up.

## Results

Seven unrelated Han Chinese patients (four boys and three girls) were enrolled in the present study. None of these patients were from parental consanguineous families, whereas two patients had a positive family history; the older sister of patient 1 died due to renal failure at the age of 3 years, and the older sister of patient 4, who died at the age of 28 months, had a history of pulmonary arterial hypertension and development delay.

The onset age of the seven patients ranged from 9 months to 5 years. Time from onset to diagnosis varied from 1 month to 6.25 years. Late diagnosis appeared to be associated with the long-term prognosis of the disease, as both cases 2 and 5 progressed to chronic kidney disease (CKD). Two cases first presented with edema and nephrotic syndrome (patients 3 and 7), and microscopic hematuria and proteinuria were detected accidentally in two cases (patients 1 and 2). At disease onset, patient 4 manifested with edema, gross hematuria, and proteinuria after infection, associated with macrocytic anemia; patient 5 presented with proteinuria without any treatment; and patient 6 had acute renal failure after infection, accompanied by heart failure, and was treated with continuous renal replacement therapy (CRRT) before admission to our hospital.

**Figure 1:**
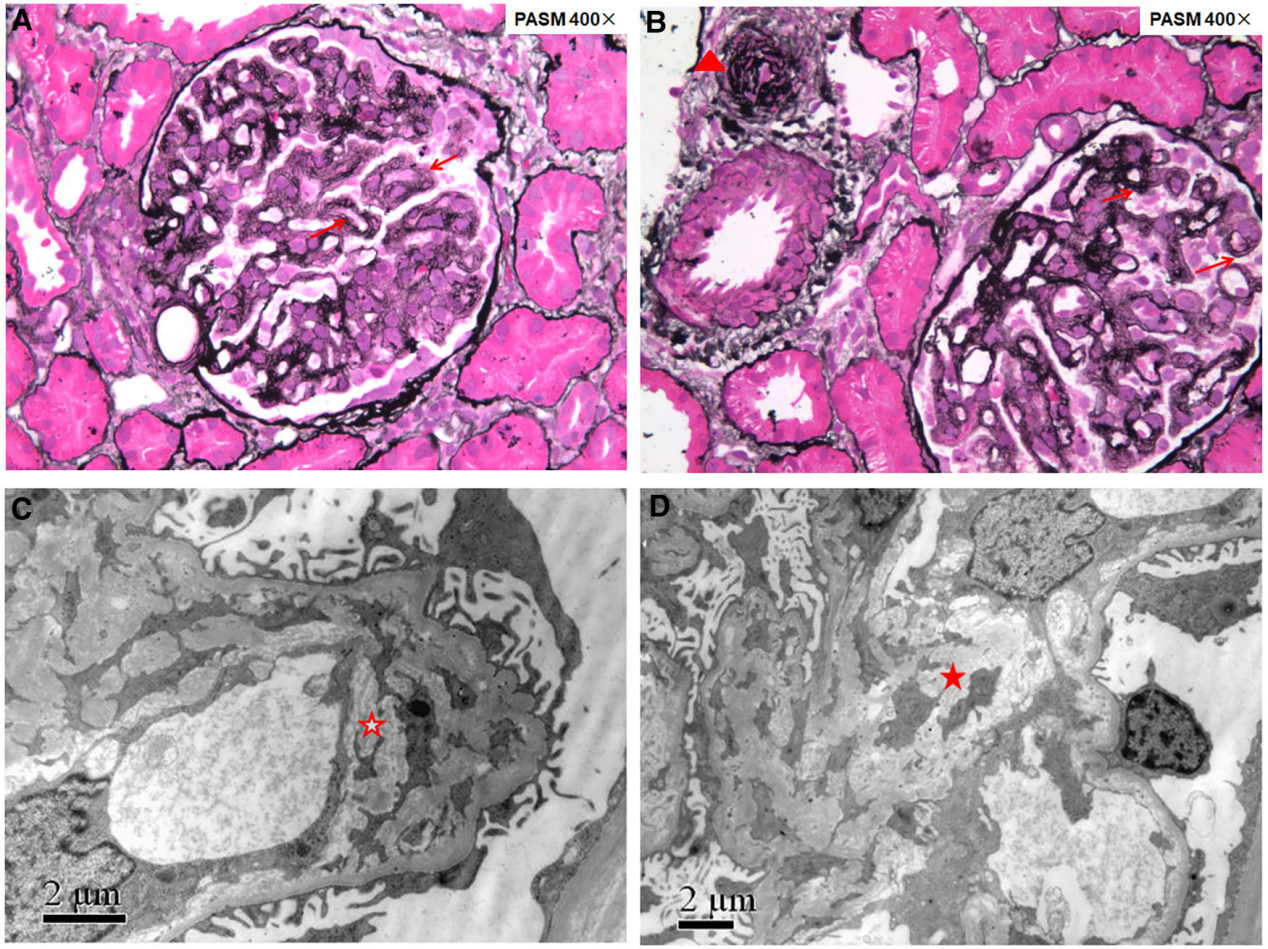
Renal pathology of patient 1 showing renal thrombotic microangiopathy. Fourteen glomeruli were sampled for light microscopy evaluation (**A,B**): the glomerular basement membrane thickened diffusely with double contours (red arrows); arterioles showed narrowed lumens with endothelial swelling and intimal onion-skin change (red triangle). Electron microscopy evaluation (**C,D**): the glomerular basement membrane thickened with subendothelial widening filled with electron-lucent fluffy material, diffusely mesangial interposition along the inner layer of the glomerular basement membrane to form a double contour (red hollow star), and electron transparent mesangial lysis can be seen in the mesangial region (red solid star).

All seven patients presented with hematuria and proteinuria. Microscopic hematuria was detected in six patients, and episodic gross hematuria (induced by infection) was observed in one patient. Nephrotic proteinuria was detected in three patients (patients 3, 4, and 7), two of whom also had edema and hypoalbuminemia and were diagnosed with nephrotic syndrome. Mild-to-moderate proteinuria was detected in the remaining four patients. Three patients had normal renal function, whereas four cases presented with renal dysfunction, including one case (case 3) giving up treatment and dying from renal failure 3 months later. Overt elevated serum level of lactate dehydrogenase (LDH) was detected in 3/7 cases. Both the total and indirect bilirubin levels were normal in all seven patients. Peripheral blood smears were performed for three cases, and the percentage of schistocytes was 0.3% in patient 2; schistocytes were observed occasionally in patient 4, and no schistocytes were observed in patient 1. Overt elevated serum level of reticulocytosis was detected in 6/7 cases; it was normal in only patient 7. Serum complement levels of C3 and C4 were normal in 6/7 cases. The levels of complement C3 and H factor were decreased in patient 5, while serum factor H autoantibody was negative. Complement H factor and factor H autoantibody were not detected in the other six patients. Renal ultrasound was performed on all patients, and the kidneys were all normal in size. Five patients underwent renal biopsy 1 month to 6 years after onset, and all had TMA (Figure 1).

All seven cases presented with extrarenal involvement. Mild-to-moderate macrocytic anemia was detected in all seven patients, whereas platelet count was normal. Six out of seven cases had hypertension, and 2/7 cases presented with pulmonary hypertension. Neurological complications were not prominent; two cases (patients 1 and 7) suffered from mild intellectual disability, and one of them suffered from epilepsy. An ocular examination was not done.

During the disease course, elevated levels of urine methylmalonic acid and plasma homocysteine were detected in all seven cases (Table 1). It is worth noting that two patients (patients 4 and 5) had normal levels of urine methylmalonic acid at the initial evaluation. Due to the young age of onset, macrocytic anemia with hematuria and proteinuria, blood vitamin B12 deficiency, and positive family history, patient 4 was considered to have cblC deficiency despite having a normal urine methylmalonic acid level. A further examination revealed elevated serum homocysteine, and finally, the diagnosis was confirmed by genetic testing. Patient 5 was initially found to have proteinuria due to increased foam in urine when she was 3 years old. One year after the onset of the disease, microscopic hematuria and nephrotic proteinuria with renal dysfunction were found. At the same time, inflammatory dilated cardiomyopathy was observed without anemia, while the urine methylmalonic acid level was normal. Macrocytic anemia (hemoglobin 103 g/L, mean corpuscular volume 119.6 fL) was found 4.5 years after onset. Increased urine methylmalonic acid and serum homocysteine levels were detected at 6.25 years after the onset, and finally, genetic testing was performed to diagnose cblC deficiency.

**Table 1:** Clinical and biochemical characteristics of the seven patients with cblC deficiency.

Characteristic	Patient 1	Patient 2	Patient 3	Patient 4	Patient 5	Patient 6	Patient 7
Gender	F	M	M	M	F	M	F
Onset age	4y	4y	16m	9m	3y	5y	4y
Time from onset to diagnosis	1m	6y	1.5m	1.5m	6.25y	1m	5m
Onset eGFR (ml/min/1.73 m^2^)	96	52	21	108	42	34	99
Hematuria	+	+	+	+	+	+	+
Proteinuria	+	+	+	+	+	+	+
Macrocytic anemia	+	+	+	+	+	+	+
Hemoglobin (g/L)	86	112	88	79	104	73	84
Platelets (×10^9^/L)	279	248	261	284	289	314	198
Reticulocyte (%)	1.99	3.30	12.8	13.5	3.38	12.46	1.15
Total bilirubin (1.7–20 µmol/L)	7.6	8.9	11.5	18.5	6.6	7	8.2
Direct bilirubin (0–6 µmol/L)	0.5	0.77	4.2	0.7	2.4	0.3	1.9
Hypertension	−	+	+	+	+	+	+
Pulmonary hypertension	−	+	−	−	+	−	−
Intellectual disability	+	−	−	−	−	−	+
Epilepsy	−	−	−	−	−	−	+
LDH (100–240 U/L)	319	293	588	620	249	1107	278
Homosysteine (5–15 µmol/L)	170	252	555	212	174	125	156
Urine MMA (0.2–3.6 mg/gCr)	15.2	119.8	13.6	2.77	0.92 at onset 42.91 at diagnosis	3.9	10.4
Renal biopsy	TMA	TMA	TMA	ND	ND	TMA	TMA
*MMACHC* variant	c.80A > G [p. (Gln27Arg)] c.658_660delAAG [p. (Lys220del)]	c.80A > G [p. (Gln27Arg)] c.609G > A [p. (Trp203*)]	c.80A > G [p. (Gln27Arg)] c.658_660delAAG [p. (Lys220del)]	c.80A > G [p. (Gln27Arg)] c.80A > G [p. (Gln27Arg)]	c.80A > G [p. (Gln27Arg)] c.609G > A [p. (Trp203*)]	c.80A > G [p. (Gln27Arg)] c.609G > A [p. (Trp203*)]	c.80A > G [p. (Gln27Arg)] c.609G > A, [p. (Trp203*)]
Follow-up duration after diagnosis	4.5y	8y	Dead	7y	7y	7y	3y
Last urine protein	Negative	3.32 g/24 h	ND	Negative	Negative (post transplantation)	Negative	Negative
Last urine RBC	Negative	Negative	ND	Negative	Negative (post transplantation)	Negative	Negative
Last eGFR (ml/min.1.73 m^2^)	97	64.84	ND	158	Kidney transplant	122.8	128
Last homosysteine (5–15 µmol/L)	88.7	75	ND	92.86	96.87	54.82	34.45
Last urine MMA (0.2–3.6 mg/gCr)	56.01	0.83	ND	61.69	0.9	0.8	2.76

F, female; M, male; y, year; m, month; eGFR, estimated glomerular filtration rate; TMA, thrombotic microangiopathy; ND, not done.

After diagnosis, hydroxocobalamin was used at 1,000 μg IM in a single daily dose for the first – 3 months, followed by hydroxocobalamin 1 mg twice per week to 20 mg per day according to the plasma homocysteine level, targeting to lower than 50 µmol/L. Meanwhile, betaine (250 mg/kg/day), L-carnitine (50–100 mg/kg/day), and calcium folinate (5–15 mg/day) were supplemented. In addition, supportive treatments were given when necessary. Patient 6 required ventilation and CRRT at disease onset. Six cases were followed up for 3–8 years, except patient 3, who died 3 months after disease onset because of giving up treatment. Macrocytic anemia was recovered after 1–1.5 months of treatments, and proteinuria was recovered after 2 months to 2 years of treatments. Both proteinuria and microscopic hematuria did not relapse at the follow-up in all patients with normal renal function, while patient 2 had persistent proteinuria. Hypertension was persistent and required treatment with oral antihypertensive medications. Renal function recovered after 1 year in two cases (patients 2 and 6), whereas patient 2 progressed to stage 2 CKD 13 years after onset. Eleven years after the initial presentation, at 15 years, patient 5′s renal function deteriorated and progressed to end-stage kidney disease, and then peritoneal dialysis was introduced. She received kidney transplantation 12 years after onset when she was 16 years old. Before peritoneal dialysis, she still had overt proteinuria and microscopic hematuria, with bilateral renal atrophy. In addition, the last plasma homocysteine level for the six patients was still high, ranging from 34.45 to 96.87 µmol/L, whereas the last urine methylmalonic acid level was normal in 4/6 cases (Table 1).

As shown in Table 1, three previously reported pathogenic variants c.80A > G [p. (Gln27Arg)], c.609G > A [p. (Trp203Ter)], and c.658_660delAAG [p. (Lys220del)] of the *MMACHC* gene (NM_015506) were identified in seven patients, four patients, and two patients, respectively. Six patients had compound heterozygous variants, and one had a homozygous variant.

## Discussion

CblC deficiency is a rare but usually treatable disease biochemically characterized by elevated plasma homocysteine accompanied by methylmalonic aciduria (6). However, renal involvement as the initial symptom of cblC deficiency is not common, and both plasma homocysteine and urine methylmalonic acid are not routinely measured; thus, cblC-associated renal disease is frequently misdiagnosed. Therefore, cblC deficiency was suggested to be screened in patients with either unclear intravascular hemolysis, hematuria, and proteinuria or renal TMA (6). In the present study, the duration from finding renal signs to the clinical diagnosis in patient 1 was 1 month due to noticeable macrocytic anemia without deficiency of vitamin B12 and folic acid; subsequently, hyperhomocysteinemia was detected, followed by an elevated urine methylmalonic acid level. In patient 4, glomerulopathy, macrocytic anemia with increased reticulocyte count and normal levels of vitamin B12 and folic acid, and elevated blood levels of LDH and homocysteine were identified successively, whereas the urine methylmalonic acid concentration was normal. Even so, cblC deficiency was strongly suspected; genetic testing revealed that the patient had *MMACHC* homozygous disease-causing variant c.80A > G inherited from his mother (his father's DNA was not available). These findings suggested that in patients with clinically presumed unknown causes of hematuria and proteinuria, macrocytic anemia and screening for the blood homocysteine level were more useful than screening for the urine methylmalonic acid level to indicate cblC defect. On the other hand, renal disease in four of seven patients completely resolved, providing evidence for the importance of timely identification and specific therapy of cblC defect (6).

HUS and renal pathology characterized by TMA were the most frequent manifestations of cblC defect-associated renal disease, and the age of onset varied from the neonatal to adult stage (2–4, 6). However, although TMA was the only kidney histologic pattern in our series, thrombocytopenia and serum markers of hemolysis with increased LDH, indirect bilirubin, and reticulocyte count were not prominent, and the preschool age of onset was most common. In the five reported Chinese cases with genetically proven child- and adolescence-onset cblC-associated renal disease (Table 2), a similar phenomenon was observed (10–14). *MMACHC* genotypes may not explain the reasons for these differences. Furthermore, the renal pathology of patient 1 (Figure 1), undergoing in 1 month after onset, revealed chronic TMA lesions such as duplication of the glomerular basement membrane and vascular onion-skin hyperplasia, which demonstrated the insidious onset of cblC-associated renal disease.

**Table 2:** Summary of previous reports of Chinese cblC deficiency with renal complications.

References	Age at onset (years)	Gender	Race/nationality	*MMACHC* variant	Renal presentation	Renal pathological diagnosis	Other manifestations	Outcomes
Chen et al. (10)	4	F	Chinese	c. 80A > G [p. (Gln27Arg)] c. 609G > A [ p. (Trp203*)]	Acute kidney injury	Membranous proliferative glomerular lesions and TMA	Severe normochromic anemia	Resolved completely
Pang et al. (11)	16	M	Chinese	c.1A > G [p. (Met1Val)] c.80A > G [p. (Gln27Arg)]	Hemolytic uremic syndrome	TMA	None	Dialysis
Chen et al. (12)	7	F	Chinese	c. 80A > G [p. (Gln27Arg)] c. 609G > A [ p. (Trp203*)]	Renal dysfunction and high blood pressure	TMA combined with focal renal cortical necrosis	Multiple organ failure	ESKD
Wang et al. (13)	17	F	Chinese	c.80A > G [p. (Gln27Arg)] c.482G > A [p. (Arg161Gln)]	Intermittent edema, proteinuria, and hematuria	Secondary MN (no EM)	None	Resolved completely
Chen et al. (14)	3.5	M	Chinese	c. 80A > G [p. (Gln27Arg)] c. 609G > A [p. (Trp203*)]	Edema, proteinuria, and hematuria	Diffuse membranoproli- ferative lesions of the glomerulus with deposition of IgM	Macrocytic anemia, frequent convulsions, and persistent hypertension	Resolved completely

F, female; M, male; TMA, thrombotic microangiopathy; ESKD, end-stage kidney disease; MN, membranous nephropathy; EM, electron microscopy.

The disease course of patient 5 demonstrated the variable phenotypes of cblC deficiency and underlined the diagnostic challenge. When she simultaneously developed inflammatory dilated cardiomyopathy and renal injury, which featured microscopic hematuria, massive proteinuria, and renal dysfunction, neither anemia nor increased urine methylmalonic acid was detected. Macrocytic anemia without deficiency of folate and vitamin B12 occurred until 4.5 years after onset; meanwhile, renal and cardiac impairment persisted. The initial normal urine methylmalonic acid level and unheeded macrocytic anemia without measurement of plasma homocysteine led to a delay in the diagnosis of cblC deficiency. In addition, the biochemical hallmarks of cbl deficiency types E and G are hyperhomocysteinemia without methylmalonic aciduria (15), whereas our study showed that normal urine methylmalonic acid levels could be observed in untreated cblC deficiency (patients 4 and 5).

Neurological involvement is considered a common phenotype in cblC deficiency. Beck et al. (5) reported that in 36 Caucasian patients with cblC-associated TMA, neurological impairment occurred more frequently in 16 with disease onset in the first 3 months of life (12/16, 75%) compared with 20 with disease onset after the age of 1 year (4/20, 20%), although *MMACHC* pathogenic variant c.271dupA was the most common genotype. However, in our study, two of the six patients with disease onset after the age of 1 year suffered from neurological impairment, seemingly higher than in Beck's study. This difference may be partly related to the limited number of patients in this study and the patients' ethnic origins.

Until now, about 130 pathogenic variants of the *MMACHC* gene have been reported (http://www.hgmd.cf.ac.uk/ac/index.php; last accessed October 29, 2022). The genotypes seem to be associated with ethnicity. The most common *MMACHC* pathogenic variants in the Caucasian population include c.271dupA, c.394C > T, and c.331C > T. Homozygosity for c.271dupA has been described in European children with an early-onset form of the disease and that for c.394C > T shows a later onset in Middle Eastern origin (7, 16). The most common *MMACHC* pathogenic variants in the Chinese population are c.609G > A, c.658_660delAAG, c.482G > A, c.80A > G, and c.567dupT (17, 18). Homozygosity for c.609G > A has been described in children with early-onset disease (19), and variant c.482G > A has been described in children with late-onset disease. Variant c.80A > G accounts for 5.95%–9.09% in all the Chinese cblC deficiency patients (17, 18), whereas in this study all seven patients carried variant c.80A > G, accounting for 57.14% (8/14) of all the mutant alleles. In addition, among five reported Chinese cases with child- and adolescence-onset cblC-associated renal disease and genetic diagnosis (10–14), all of them had *MMACHC* heterozygous variant c.80A > G (Table 2). In contrast, the most frequent damaged allele in Caucasian patients with cblC defect and renal TMA was c.271dupA (5, 6). Therefore, we speculated that Chinese dominant cblC-associated renal disease might be highly associated with *MMACHC* pathogenic variant c.80A > G.

The limitation of this study includes the limited number of patients, the absence of molecular testing of the genes associated with genetic atypical hemolytic uremic syndrome, and the lack of information about ocular manifestations (6, 20, 21). Nevertheless, to our knowledge, it was the first case series of Chinese pediatric genetically proven cblC deficiency presented predominantly with renal disease reported to date, which extended the phenotypic spectrum and would help improve the management of the disease in clinical practice.

## Conclusions

*MMACHC* variant c.80A > G may be associated with prominent renal complications in Chinese cblC patients. Macrocytic anemia and hyperhomocysteinemia are useful clues for patients with hematuria and proteinuria caused by cblC defect. Early diagnosis and treatment resulted in improving renal and hematological signs.

## Data Availability

The original contributions presented in the study are included in the article/Supplementary Material, further inquiries can be directed to the corresponding author/s.
